# Drug-Resistance Biomarkers in Patient-Derived Colorectal Cancer Organoid and Fibroblast Co-Culture System

**DOI:** 10.3390/cimb46060346

**Published:** 2024-06-11

**Authors:** Kyoung-Bin Ryu, Jeong-ah Seo, Kyerim Lee, Juhyun Choi, Geon Yoo, Ji-hye Ha, Mee Ryung Ahn

**Affiliations:** Clinical Research Division, National Institute of Food and Drug Safety Evaluation, Ministry of Food and Drug Safety, Cheongju 28159, Chungcheongbuk-do, Republic of Korea; lightbean1126@gmail.com (K.-B.R.);

**Keywords:** drug resistance, tumor microenvironment, co-culture, cancer-associated fibroblast, interferon-alpha/beta signaling, major histocompatibility complex class II

## Abstract

Colorectal cancer, the third most commonly occurring tumor worldwide, poses challenges owing to its high mortality rate and persistent drug resistance in metastatic cases. We investigated the tumor microenvironment, emphasizing the role of cancer-associated fibroblasts in the progression and chemoresistance of colorectal cancer. We used an indirect co-culture system comprising colorectal cancer organoids and cancer-associated fibroblasts to simulate the tumor microenvironment. Immunofluorescence staining validated the characteristics of both organoids and fibroblasts, showing high expression of epithelial cell markers (EPCAM), colon cancer markers (CK20), proliferation markers (KI67), and fibroblast markers (VIM, SMA). Transcriptome profiling was conducted after treatment with anticancer drugs, such as 5-fluorouracil and oxaliplatin, to identify chemoresistance-related genes. Changes in gene expression in the co-cultured colorectal cancer organoids following anticancer drug treatment, compared to monocultured organoids, particularly in pathways related to interferon-alpha/beta signaling and major histocompatibility complex class II protein complex assembly, were identified. These two gene groups potentially mediate drug resistance associated with JAK/STAT signaling. The interaction between colorectal cancer organoids and fibroblasts crucially modulates the expression of genes related to drug resistance. These findings suggest that the interaction between colorectal cancer organoids and fibroblasts significantly influences gene expression related to drug resistance, highlighting potential biomarkers and therapeutic targets for overcoming chemoresistance. Enhanced understanding of the interactions between cancer cells and their microenvironment can lead to advancements in personalized medical research..

## 1. Introduction

Colorectal cancer (CRC) is the third most prevalent cancer worldwide, only after breast and lung cancers, and is the second leading cause of cancer-related mortality, succeeded by lung cancer [1]. While the incidence of CRC has decreased among individuals aged 65 and older in most countries [2], a concerning rise has been reported among those aged under 50 worldwide [3]. Nearly a quarter of patients with CRC present with metastatic disease [4]. The median overall survival of patients with metastatic CRC is approximately 20 months [5], emphasizing the need for improved therapeutic strategies.

A major challenge in treating CRC is the development of drug resistance, which severely limits the effectiveness of chemotherapeutic agents. The mechanisms underlying drug resistance are complex and multifaceted, involving alterations in drug uptake and efflux, changes in drug target sensitivity, DNA damage repair mechanisms, and interactions with the tumor microenvironment (TME). Understanding these mechanisms is crucial for developing new strategies to overcome resistance and improve patient outcomes.

CRC progresses in distinct ways, each dictating specific treatment modalities, including surgery, chemotherapy, or radiation therapy [6,7,8,9]. Chemotherapy, particularly with 5-fluorouracil (5-FU), irinotecan, or oxaliplatin, is pivotal. 5-FU has been the most effective anticancer agent for over 40 years, and the FOLFOX regimen combining oxaliplatin, 5-FU, and leucovorin has been explored as a first-line treatment for advanced colorectal cancer [10]. However, CRC quickly develops resistance to FOLFOX, resulting in significant toxicity and patient morbidity [11]. Variable efficacy and post-treatment drug resistance remain challenging [12], necessitating a deeper understanding of the underlying mechanisms.

Fibroblasts and their activated counterparts residing inside the tumor mass, namely cancer-associated fibroblasts (CAFs), are enigmatic cells. CAFs show increased proliferation and motility and elevated secretion of growth factors, chemokines, and extracellular matrix (ECM)-degrading enzymes such as metalloproteases, compared to normal fibroblasts. Thus, CAFs regulate tumorigenesis and metastasis positively [13,14]. CAFs contribute to the generation and maintenance of the cancer stem cell ‘niche’ through an active remodeling of the ECM and secretion of morphogens [15,16]. CAFs interact with tumor cells and are functionally connected with other cells in the tumor microenvironment (TME), including vascular endothelial cells and immune cells. CAFs secrete factors that modulate vascular network formation/remodeling [17,18,19] and affect the function of several immune cell types, including macrophages, neutrophils, and T cells [20]. Several research groups have reported that CAFs promote the formation of an immunosuppressive environment directly through the secretion of several chemokines or other negative immune regulators [21,22] and indirectly by regulating the stiffness of the ECM, which decreases immune cell infiltration or immune cell extravasation [23].

CAF-mediated tumor–stroma interactions, particularly prevalent in the CRC TME, are pivotal in tumor progression, affecting cell proliferation, stem cell regulation, and drug resistance [24,25]. Recent advances in co-cultures of patient-derived organoid (PDO) models with CAFs have revealed sustained proliferation within hydrogels, emphasizing the suitability of this model for drug evaluations for CRC treatment [26].

Effects of CAFs on tumor organoids and transplantation xenograft models for hepatocellular carcinoma underscores their role in promoting tumor growth and conferring drug resistance [27]. Similarly, studies on pancreatic ductal adenocarcinoma highlight the critical role of CAFs in drug resistance [28].

Interactions within the TME, including tumor cells, microenvironment-specific stromal cells (CAFs), and the ECM, significantly affect tumor growth and drug responses [29,30]. Traditional two-dimensional in vitro techniques fail to model the complexity of the TME accurately, prompting the exploration of innovative three-dimensional culture systems, such as organoids [31,32,33].

This study employed an indirect co-culture system to mimic the TME, and RNA transcriptome analysis was conducted on samples treated with specific anticancer drugs. The objective of this study was to identify biomarkers associated with anticancer drug resistance to advance our understanding of the intricate mechanisms underlying drug responses in the context of CRC.

## 2. Materials and Methods

### 2.1. Establishment of Patient-Derived Organoids and CAFs

In this study, we conducted experiments using CAFs and organoids derived from a single patient to explore the changes induced by co-culturing CAFs with organoids within the homogenous biological system. (The patient was a 91-year-old female with stage IIIc cancer and had no prior drug treatments. We selected this patient to ensure that this study’s results were solely due to the chemotherapy treatment applied in this research, without confounding effects from previous treatments.) To prepare the organoids, we first sectioned the tissue into fine fragments, thoroughly washing them in a solution of 70% ethanol and ice-cold PBS (PBS; Catalog No. 10010023, Thermo Fisher Scientific, Waltham, MA, USA) containing 3% Penicillin/Streptomycin (15140122, Thermo Fisher, Waltham, MA, USA), repeating the process a minimum of five times. The tissue was then finely chopped into 10 cm samples using surgical forceps and a scalpel or scissors. The finely diced tissue samples were then immersed in a digestion medium consisting of DMEM, supplemented with 1% Penicillin/Streptomycin, 2.5% Fetal Bovine Serum (FBS;10082147, Thermo Fisher, Waltham, MA, USA), collagenase type IV (17104029, Thermo Fisher, Waltham, MA, USA) and 125 μg/mL Dispase II (17105041, Thermo Fisher, Waltham, MA, USA). This mixture was incubated for 1 to 2 h at a temperature of 37 °C. Post-digestion, the samples were centrifuged to form a pellet, followed by a resuspension in fresh DMEM. The suspension was then filtered through a 40 μm cell strainer (352340, Corning, Bedford, MA, USA) and centrifuged at 1000 rpm for 5 min at a temperature of 4 °C. Subsequently, the pellet was resuspended in DMEM and centrifuged again at 3000 rpm for 1 min at 4 °C to eliminate any residual debris and collagenase. The final cell pellet was then mixed with Matrigel (354230, Corning, Bedford, MA, USA) and carefully allocated into 48-well culture plates, using 25 μL of Matrigel for each well. Organoid culture media was formulated with Advanced DMEM/F-12 (Gibco, Carlsbad, CA, USA), HEPES (Gibco, Carlsbad, CA, USA), Penicillin–Streptomycin (Gibco, Carlsbad, CA, USA), GlutaMAX (Gibco, Carlsbad, CA, USA), and FBS (Gibco, Carlsbad, CA, USA), supplemented with niche factors such as N-Acetyl-L-cysteine, Nicotinamide, N-2 Supplement, B-27 Supplement, Gastrin, SB202190, A83-01, hEGF, Human Noggin, Human R-Spondin-1, and Y-27632 to promote growth and differentiation.

To prepare the CAFs from CRC, tissue samples are first sectioned into 0.5 cm^2^ pieces, decontaminated with two washes in 80% ethanol, and then rinsed three times in PBS. These pieces are finely minced to increase the surface area for enzymatic digestion in DMEM/F12 media containing 0.1% collagenase and are incubated at 37 °C in a rotating incubator for 4–8 h to facilitate tissue dissociation. Following digestion, the cell mixture is transferred to DMEM/F12 media supplemented with 10% FBS and cultured in a 60 mm dish. The medium conditions and organoid preparation methods are modified from Lee et al. [34,35].

### 2.2. Co-Culture and Drug Treatment

Organoids were cultured in a 24-well Transwell plate (3413, Thermo Fisher, Waltham, MA, USA), with fibroblasts cultured in the insert of the Transwell plate featuring a pore size of 0.4 µm to allow free media exchange without cell migration. One day prior to co-culture initiation, fibroblasts were dissociated into single cells using TrypLE Express (12605036, Thermo Fisher, Waltham, MA, USA), and 2.4 × 10^4^ cells were seeded in the insert. For organoid culture, cells were set on the 24-well plate with 20 µL of Matrigel polymerized. Dissociated organoids were resuspended in optimized media, and the cell suspension (6 × 10^3^/20 µL) was seeded onto Matrigel, with 500 µL of organoid media added to the basal compartment. The number of CAFs was based on the transwell size, and preliminary co-culture studies (CAF/Organoid; 0.5:1, 1:1, 2:1, 4:1, 8:1) showed that the 4:1 (CAF/Organoid) ratio was most optimal for growth. The passage number of the fibroblasts used was 10. Fibroblasts and organoids were incubated at 37 °C in a CO_2_ incubator.

The following day, apical and basal media were removed from the organoid and fibroblast plate. The fibroblast-containing inserts were transferred to the organoid-containing compartment after changing the organoid media to 1500 µL of co-culture media (Advanced DMEM/F-12, 10 mM HEPES, 1% Penicillin–Streptomycin, 1X GlutaMAX, 5% FBS). The co-culture plate was incubated at 37 °C in a CO_2_ incubator for 120 h, with organoids and fibroblasts collected separately for gene expression analysis. Drug treatments with 5-FU and oxaliplatin were administered at concentrations of 5 µM and 2 µM, respectively, according to the dose-dependent IC50 values identified in separate assays that took into account CAF survival using WST-8 and Cell Titer-Glo 3D reagent (Promega Corporation^®^, Madison, WI, USA) to ensure a balanced approach to chemoresistance studies. Drugs were treated in co-culture media and administered simultaneously with the start of co-culture.

### 2.3. Immunofluorescence Staining

Specimens were fixed in 4% paraformaldehyde in PBT (phosphate-buffered saline + 0.1% Tween-20) overnight at 4 °C, followed by thorough rinsing in PBT. For immunostaining, fixed samples underwent a 2 h incubation in blocking solution (Sigma, St. Louis, MO, USA), followed by additional rinses in PBT. After several PBT washes, samples were preincubated for 30 min at room temperature in PBT, then incubated overnight at 4 °C in primary antibodies (mouse anti-alpha smooth muscle Actin, ab7817, Abcam; mouse anti-EPCAM, #2929, Cell Signaling Technology, Danvers, MA, USA; rabbit anti-KI67, ab16667, Abcam; rabbit anti-Cytokeratin 20, ab76126, Abcam; anti-Vimentin, ab137321, Abcam, Cambridge, UK) dissolved at a 1:200 ratio in PBT.

Following several PBT rinses, samples were preincubated for an additional 30 min at room temperature in PBT, followed by overnight incubation at 4 °C in secondary antibodies (goat anti-mouse conjugated with AlexaFluor488, ab150113, Abcam, Cambridge, UK; or AlexaFluor568, A11011, Invitrogen, Waltham, MA, USA) at a 1:1000 dilution in PBT. Subsequently, the specimens underwent multiple rinses in PBT and were stained for cell nuclei with DAPI (4′,6-Diamidino-2-phenylindole dihydrochloride, 1:500 solution in PBS) for 1 h. The protocol conditions for immunofluorescence staining were modified from those described in Han et al. [36].

### 2.4. RNA Sample Preparation and Library Construction

Total RNA was extracted from six distinct samples, each processed in triplicate to ensure the reliability and reproducibility of our findings. The samples included monoculture organoid control (M_Ctrl), monoculture organoid treated with 5-FU (M_5FU), monoculture organoid treated with oxaliplatin (M_Oxa), co-culture organoid control (C_Ctrl), co-culture organoid treated with 5-FU (C_5FU), and co-culture organoid treated with oxaliplatin (C_Oxa), using Trizol reagent (Invitrogen, Waltham, MA, USA). RNA concentrations were determined using Quant-IT RiboGreen (Invitrogen, Waltham, MA, USA). To assess RNA integrity, samples were analyzed on TapeStation RNA screentape (Agilent Technologies, Santa Clara, CA, USA), and only samples with an RNA Integrity Number (RIN) greater than 7.0 were utilized for library construction.

The RNA isolated from each sample was used to construct sequencing libraries with the SMART-Seq^®^ mRNA Kit (634894, Takara bio, Mountain View, CA, USA), following the manufacturer’s protocol. First-strand cDNA synthesis, primed by the 3′ SMART-Seq CDS Primer II A, utilized the SMART-Seq v4 Oligonucleotide for template switching at the 5′ end of the transcript. Magnetic separation with SPRI beads was employed to selectively bind first-strand cDNA, removing contaminants from the solution. The beads were then directly utilized for PCR amplification, employing the Advantage 2 Polymerase Mix for efficient and accurate long-distance PCR amplification of cDNA templates. PCR-amplified cDNA was purified using AMPure XP beads, washed with 80% ethanol, and eluted with Elution Buffer.

Sequencing libraries were constructed following the Nextera XT DNA Library Preparation Kit instructions (Illumina, San Diego, CA, USA). Briefly, 1 ng of cDNA underwent tagmentation for DNA fragmentation and adapter tagging in a single step. Indexes were attached through PCR using Nextera-indexed primers. The final purified product was quantified using qPCR (KAPA Library Quantification kits for Illumina Sequencing platforms) and qualified using TapeStation HS D5000 ScreenTape (Agilent Technologies, Santa Clara, CA, USA). Paired-end (2 × 100 bp) sequencing was performed by Macrogen using the NovaSeq platform (Illumina, San Diego, CA, USA).

### 2.5. Data Processing and Analysis

Paired-end sequencing reads were obtained using the Illumina NovaSeq platform. Before starting the analysis, Trimmomatic v0.38 was used to remove adapter sequences and trim bases with poor base quality. The resultant cleaned reads were aligned to the Homo sapiens (GRCh38) reference genome using HISAT v2.1.0 [37], leveraging HISAT and Bowtie2 implementations. The reference genome sequence and gene annotation data were acquired from the NCBI Genome assembly and NCBI RefSeq database, respectively. Aligned data in SAM file format were sorted and indexed using SAMtools v1.9. Following alignment, transcripts were assembled and quantified using StringTie v2.1.3b [38,39] Gene-level and transcript-level quantification metrics, including raw read count, FPKM (fragments per kilobase of transcript per million mapped reads), and TPM (transcripts per million), were calculated. Subsequently, differential gene expression analysis was conducted using the DESeq2 package v3.19 in R. Six identified differentially expressed genes (DEGs) were subjected to gene ontology (GO) analysis using the R package clusterProfiler v4.6.2 [40]. Heatmaps were generated using Morpheus softwarev6.3.1 from the Broad Institute (https://software.broadinstitute.org/morpheus/; accessed on 23 July 2023), with datasets hierarchically clustered based on 1 minus Pearson’s correlation coefficient. Venn diagrams were constructed using Venny v2.1.0 (https://bioinfogp.cnb.csic.es/tools/venny/; accessed on 2 Aug 2023).

### 2.6. Enrichment Analysis

Metascape (http://metascape.org; accessed on 20 Aug 2023) has been identified as a useful tool for gene annotation as well as enrichment analysis of gene lists [41]. This program helps you make informed decisions based on functional gene annotations and protein lists. We utilized Metascape to perform pathway and process enrichment analysis of specific similar genes. Terms including biological processes, cellular components, and molecular functions were enriched through GO domains using the Metascape online tool. Significant terms were set when they represented a minimum overlap of 3 (*p*-value cutoff 0.05) and a minimum enrichment of 3. The results derived were considered significant. Databases such as InWeb_IM [42], OmniPath [43], BioGrid [44], and STRING [45,46] were utilized for protein–protein interaction (PPI) enrichment analysis.

Table 1 summarizes the critical points of drug-resistance biomarkers in the patient-derived colorectal cancer organoid and fibroblast co-culture system.

## 3. Results

### 3.1. Morphological Analysis and Characteristics of Organoids and CAFs

**Figure 1:**
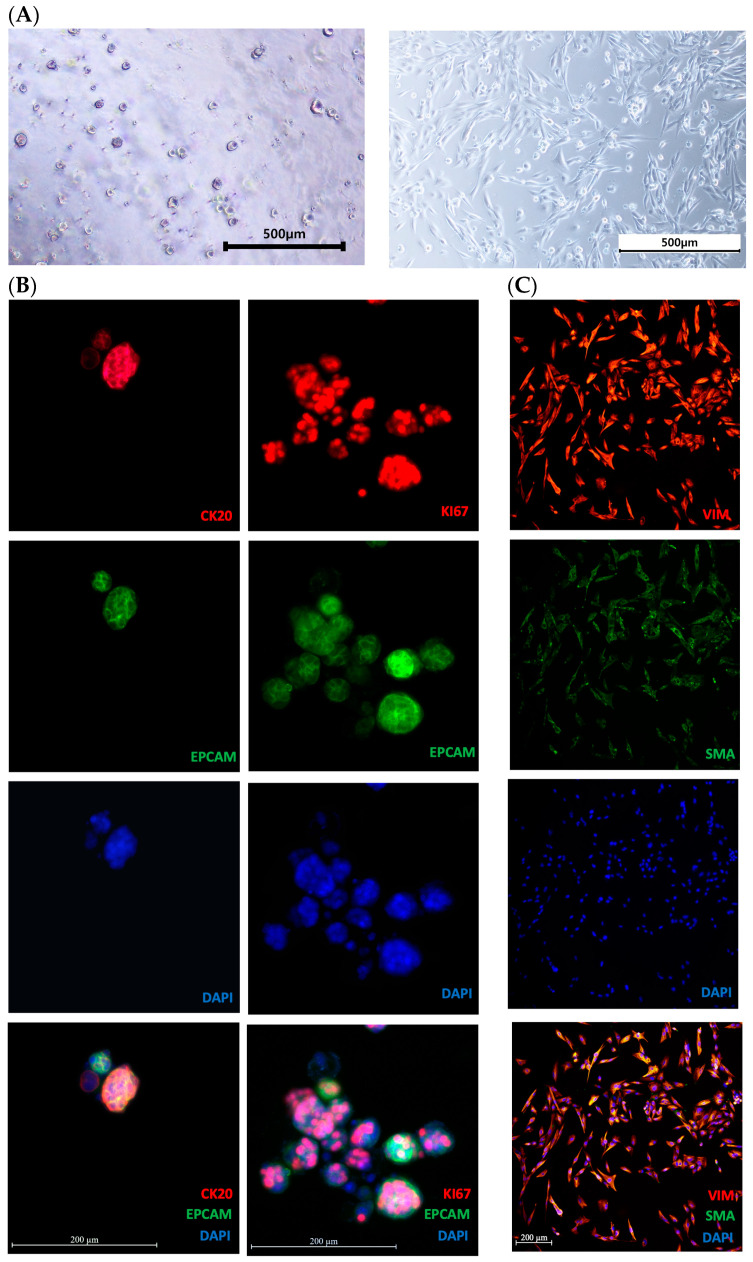
Morphological analysis by bright-field images and immunofluorescence (IF) staining with colorectal cancer markers (CK20, KI67, EPCAM) and fibroblast markers (VIM, SMA). (**A**) Bright-field images of PDO and CAF. (**B**) IF staining of CRC PDO with CK20, EPCAM, KI67, and DAPI (DNA) as indicated. (**C**) IF staining of CRC patient-derived CAF with VIM, SMA, and DAPI (DNA) as indicated. Images were taken with a Zeiss LSM 700 confocal microscope(Zeiss, Jena, Germany). Scale bars indicate 500 µm in bright-field images and 200 µm in IF images. The images confirm the preservation of original characteristics in both organoids and CAFs, validating the suitability of these samples for further experimental use.

We conducted morphological assessments using bright-field and immunofluorescence staining, utilizing biomarkers, including epithelial cell markers (EPCAM), colon cancer markers (cytokeratin 20, CK20), proliferation markers (KI67), and fibroblast markers (alpha-smooth muscle actin, SMA; vimentin, VIM) (Figure 1A–C) to validate the preservation of characteristics in organoids and CAFs. Bright-field imaging for the confirmation of morphological characteristics showed that the structures of the organoids and CAFs effectively retained their respective features (Figure 1A). Immunofluorescence staining analysis revealed high expression of EPCAM, predominantly membrane-localized in the organoids. CK20 localization, specific to CRC cells, was observed in the cytoplasm and cell membranes (Figure 1B). KI67 staining of the organoids confirmed its restricted expression in the nucleus, indicating active proliferation (Figure 1B). For CAFs, the positive expression of fibroblast markers (SMA and VIM) validated the preservation of fibroblast characteristics (Figure 1C). The overall morphological analysis confirmed the suitability of our samples (organoids and CAFs) for experimental use.

### 3.2. Co-Culture and Transcriptome Profiling

**Figure 2:**
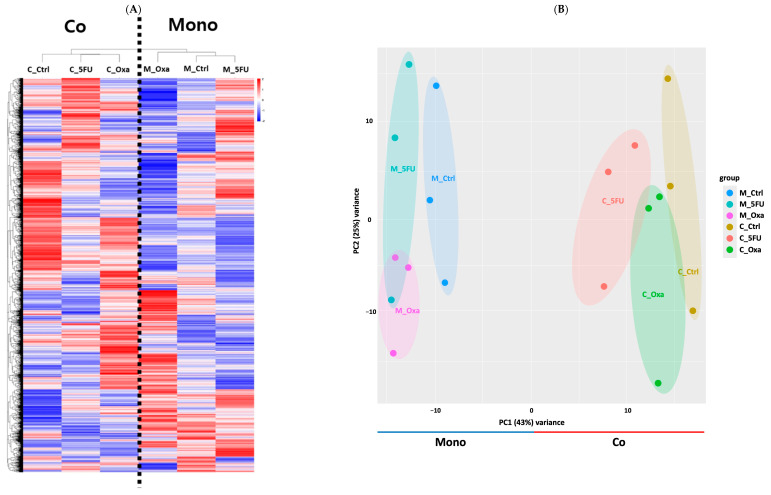
Next generation sequencing (NGS) and gene expression pattern of mRNA seq results. (**A**) Heatmap showing the expression levels of genes in six samples of organoids. Hierarchical cluster analysis revealed distinct cluster formations, highlighting a clear differentiation between monoculture organoids and co-culture organoids. (**B**) Principal component analysis of each sample. The same color indicates the number of repetitions of the experiment, and the experiment was conducted in triplicate per sample. C_Ctrl, co-culture organoid control; C_5FU, co-culture organoid treated with 5FU; C_Oxa, co-culture organoid treated with oxaliplatin; Co, co-culture organoid control; M_Ctrl, monoculture organoid; M_5FU, monoculture organoid treated with 5FU; M_Oxa, monoculture organoid treated with oxaliplatin; Mono, monoculture organoid.

In transcriptome analysis, we focused on evaluating the overall mRNA expression levels (FPKM) of 46,428 genes across six samples, including mono- and co-cultured organoids treated with control, 5-FU, or oxaliplatin to understand the differences between co-culture and monoculture conditions. Hierarchical clustering analysis revealed a significant grouping of co-culture samples, indicating a distinct transcriptomic profile compared to that of the monoculture (Figure 2A). Principal component analysis (PCA) supported these findings by demonstrating a clear separation between mono- and co-culture samples in the majority of the organoid datasets (Figure 2B).

### 3.3. DEG Profiling in CRC Organoids in a Co-Culture

**Figure 3:**
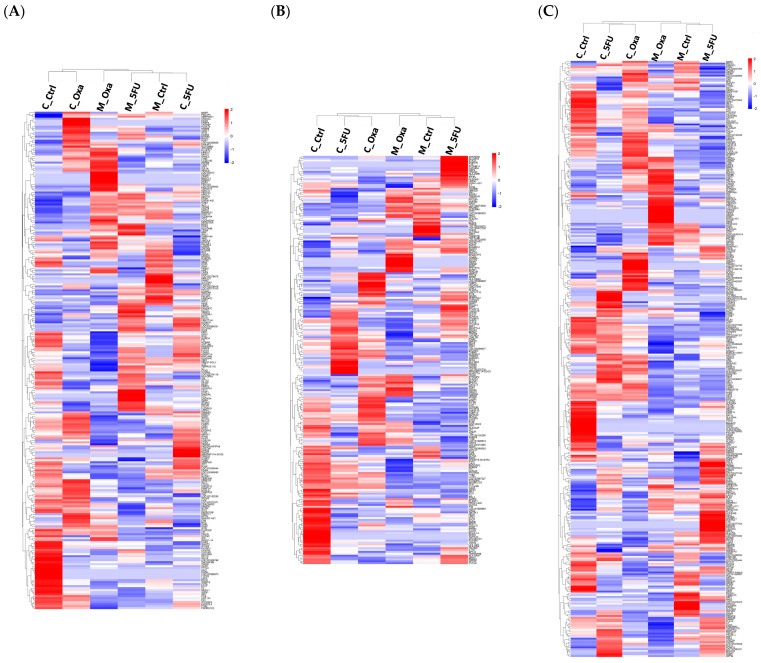

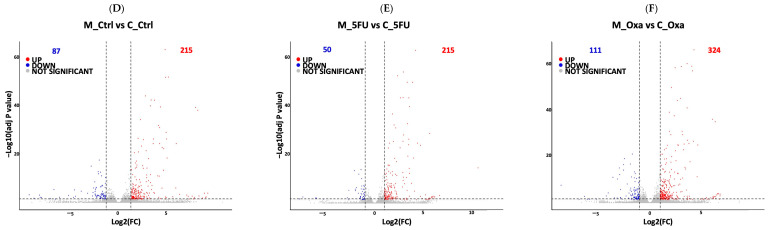
Differentially expressed genes (DEGs) analysis of each organoid sample. Hierarchical clustering heatmaps of significant genes in (**A**) M_Ctrl vs. C_Ctrl, (**B**) M_5FU vs. C_5FU, and (**C**) M_Oxa vs. C_Oxa. Volcano plots of significant genes in (**D**) M_Ctrl vs. C_Ctrl, (**E**) M_5FU vs. C_5FU, and (**F**) M_Oxa vs. C_Oxa. Red indicates upregulation, and blue indicates downregulation. Cut off (dotted line) drawn at equivalent of adjusted *p* = 0.05 and ${log}_{2}$(fold change) of 1. The hierarchical clustering heatmaps and volcano plots provide a detailed visualization of the gene expression differences between co-culture and monoculture conditions under different treatments, highlighting significant DEGs that may be involved in chemoresistance mechanisms. C_Ctrl, co-culture organoid control; C_5FU, co-culture organoid treated with 5FU; C_Oxa, co-culture organoid treated with oxaliplatin; M_Ctrl, monoculture organoid; M_5FU, monoculture organoid treated with 5FU; M_Oxa, monoculture organoid treated with oxaliplatin.

The heatmap represents the expression of all DEGs, with red indicating upregulation and blue indicating downregulation (Figure 3A–C). In the heatmap of genes differentially expressed in the co-culture organoid compared to the monoculture organoid, the monoculture and co-culture groups were not distinct (Figure 3A), unlike in the heatmap of overall expression (Figure 2B). In contrast, the heatmap of genes differentially expressed in the co-culture compared to the monoculture treated with 5FU or oxaliplatin confirmed that the monoculture and co-culture groups were separate (Figure 3B,C). Simultaneously, the volcano plot illustrated the fold change (${log}_{2}$) against statistical significance (−${log}_{10}$ *p*-value), with significant genes shown in red. These plots offer a comprehensive visualization of gene expression patterns, facilitating the identification of genes responsive to the co-culture conditions. Differential gene analysis, based on the criteria of ${log}_{2}\;$fold change > |1| and *p* < 0.05, revealed significant alterations in gene expression. A total of 302 genes were identified between the monoculture organoid and co-culture organoid groups (Figure 3D). Similarly, between the monoculture organoids treated with 5FU and the co-culture organoids treated with 5FU groups, 265 genes were differentially expressed, with 215 upregulated and 50 downregulated genes (Figure 3E). A total of 435 genes were differentially expressed between the monoculture organoids treated with oxaliplatin and the co-culture organoids treated with oxaliplatin groups (Figure 3F). More upregulated genes were identified in the overall DEG data (Figure 3).

### 3.4. Functional Enrichment Analysis of the Significant Genes in the Co-Culture

**Figure 4:**
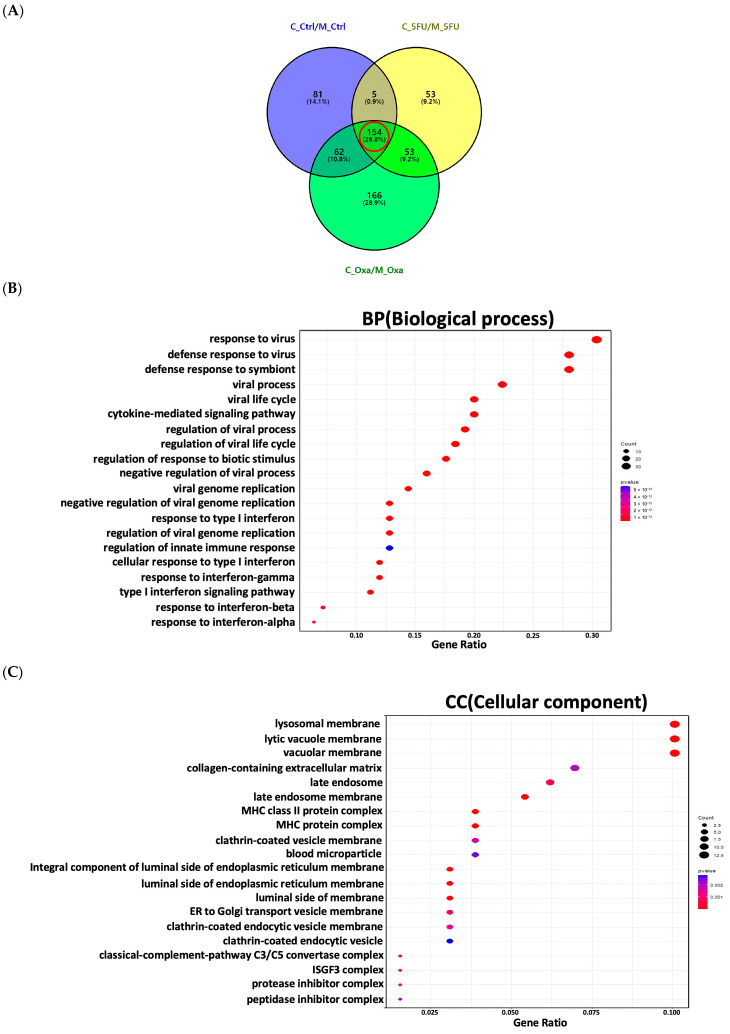

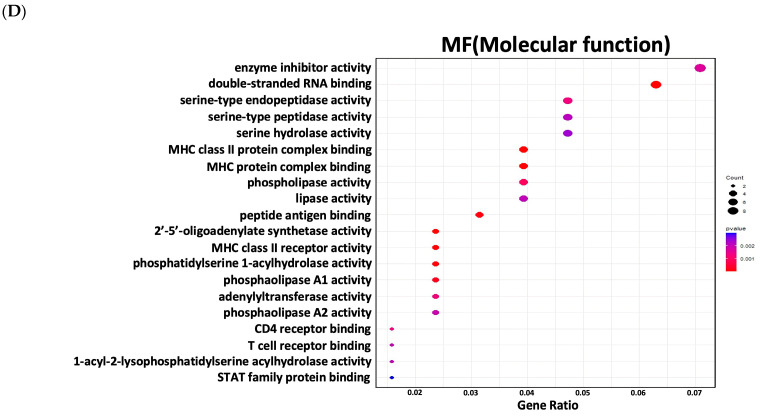
Significant genes in co-culture organoids compared to monoculture organoids by differential gene expression analysis. (**A**) Venn diagram depicting significant genes in co-culture organoids compared to monoculture organoids, identified based on the criteria of adjusted *p*-value < 0.05 and ${log}_{2}$ fold change > |1| in each sample. A total of 154 significant genes (red circle) exhibiting altered expression during co-culture organoids in comparison to monoculture organoids were identified. (**B**) Enrichment analysis of GO biological process. (**C**) Enrichment analysis of GO cellular component. (**D**) Enrichment analysis of GO molecular function. The statistical significance was evaluated with an adjusted *p* < 0.05.

In total, 302 genes were significantly differentially expressed between co-culture and monoculture organoid controls; 265 genes were significantly differentially expressed between co-culture and monoculture organoids treated with 5-FU; and 435 genes were significantly differentially expressed between co-culture and monoculture organoids treated with oxaliplatin. A total of 154 genes with commonly altering expression during co-culture were selected based on an adjusted *p*-value of less than 0.05 and a fold change (${log}_{2}$) of more than |1| (Figure 4A) to select significant genes under co-culture conditions. Enrichment analysis was performed on 154 genes following selection from the Venn diagram to identify the GO categories from a specific gene list for functional prediction. GO terms were classified into the following subcategories: biological processes (Figure 4B), cellular components (Figure 4C), and molecular functions (Figure 4D). The main enriched terms included defense response to symbionts, cytokine-mediated signaling pathway, regulation of response to biotic stimulus, response to type I interferon, regulation of innate immune response, late endosome membrane, MHC protein complex, clathrin-coated vesicle membrane, ER to Golgi transport vesicle membrane, clathrin-coated endocytic vesicle, and MHC class II protein complex binding.

### 3.5. Candidate Gene Selection

**Figure 5:**
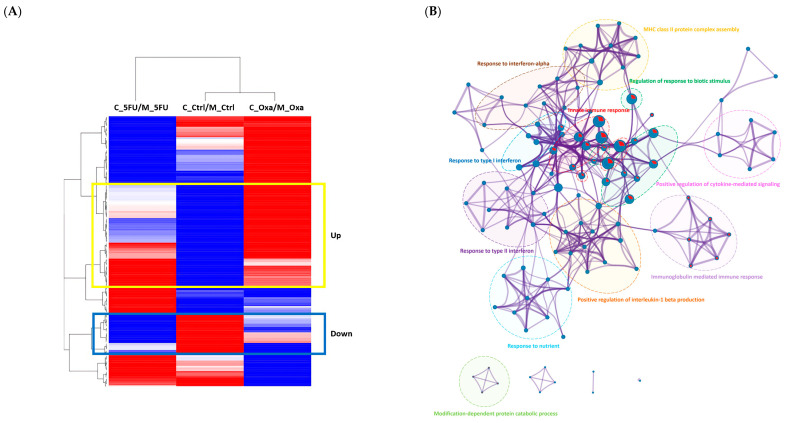

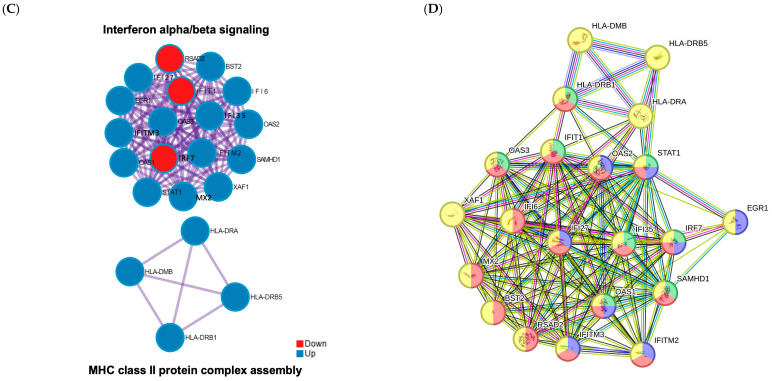
Potential drug resistance genes among 154 co-culture significant genes. (**A**) Hierarchical clustering heatmap of 154 co-culture significant genes. Yellow box indicates commonly upregulated genes, and blue box indicates commonly downregulated genes. Red indicates upregulation, and blue indicates downregulation. (**B**) GO enrichment analysis of up- and downregulated genes. Red pie indicates downregulated genes, and blue pie indicates upregulated genes. (**C**) Selected groups of protein–protein interaction (PPI) analysis by physical affinity (STRING physical score > 0.132). (**D**) Functional enrichment analysis of selected genes by STRING. Pathways represent GO: 0006952, defense response (red spheres); GO: 0050896, response to stimulus (yellow spheres); GO: 0002831, regulation of response to biotic stimulus (green spheres); and GO: 0019221, cytokine-mediated signaling pathway (blue spheres).

Hierarchical cluster analysis was performed on 154 significant co-culture genes to select those that potentially conferred drug resistance. We performed selection and comparative analyses of 58 genes that were commonly upregulated and 24 that were downregulated in the anticancer drug (5-FU, oxaliplatin) treatment groups compared to the control (Figure 5A). Enriched terms included MHC class II protein complex assembly, response to interferon-alpha, regulation of response to biotic stimulus, innate immune response, response to type I interferon, positive regulation of cytokine-mediated signaling, response to type II interferon, positive regulation of interleukin-1 beta production, immunoglobulin-mediated immune response, response to nutrients, and modification-dependent protein catabolic process. Among them, the enriched terms that overlapped with the upregulated and downregulated genes were innate immune response, regulation of response to biotic stimulus, and immunoglobulin-mediated immune response (Figure 5B). Comparative analysis of upregulated gene clusters and downregulated gene clusters from the PPI revealed two groups with high physical affinity (STRING physical score > 0.132): interferon-alpha/beta signaling (17 genes) and MHC class II protein complex (4 genes) assembly groups (Figure 5C). STRING analysis identified the following enriched pathways: defense response (Gene Ontology database [GO]:0006952, FDR, false discovery rate = 5.48 × $10^{-11}$, including BST2, *HLA-DRB1, IFI6, IFI27, IFI35, IFIT1, IFITM2, IFITM3, IRF7, MX2, OAS1, OAS2, OAS3, RSAD2, SAMHD1, STAT1*); response to stimulus (Gene Ontology database [GO]:0050896, FDR = 2.04 × $10^{-6}$, including *BST2, EGR1, HLA-DMB, HLA-DRA, HLA-DRB1, HLA-DRB5, IFI6, IFI27, IFI35, IFIT1, IFITM2, IFITM3, IRF7, MX2, OAS1, OAS2, OAS3, RSAD2, SAMHD1, STAT1, XAF1*); regulation of response to biotic stimulus (Gene Ontology [GO]: 0002831, FDR = 1.38 × $10^{-6}$, including *HLA-DRB1, IFI35, IFIT1, IRF7, OAS1, OAS3, SAMHD1, STAT1*); and cytokine-mediated signaling pathway (Gene Ontology [GO]: 0019221, FDR = 1.58 × $10^{-6}$, including *EGR1, IFI27, IFITM2, IFITM3, IRF7, OAS1, OAS2, STAT1*) (Figure 5D).

## 4. Discussion

Although the mortality rate of patients with CRC has decreased owing to advancements in medicine involving surgery and targeted drugs, problems related to the TME, such as metastasis and drug resistance, remain [47,48,49].

Interactions between tumor cells and various stromal components within the TME are emerging as key factors affecting tumor growth and metastasis [48]. Among these components, CAFs, the predominant stromal cell type in the TME, exert their effects by remodeling the ECM and promoting tumor cell progression through the secretion of diverse chemokines, cytokines, and growth factors [50].

In this study, we established a co-culture system comprising CAFs and PDOs. Utilizing next-generation sequencing (NGS) and bioinformatics approaches, we identified potential drug-resistance biomarkers. This holistic approach sheds light on the intricate interplay within the TME and provides insight into the mechanisms that may contribute to drug resistance in CRC.

The co-culture system demonstrated significant differences in gene expression compared to monoculture conditions. While various factors, such as drug treatment and mechanisms, may contribute to this expressional variance, the predominant factor may be a shift from a monoculture to a co-culture system.

Following functional analysis of genes specifically altered in the co-culture, terms associated with defense, cytokine response, stimulus-response, immune regulation, endosome activity, transport, and MHC class were analyzed. Co-culture with CAFs significantly affected the immune system, growth, and defense mechanisms of organoids [51,52]. Genes related to endosome, vesicle, and transport functions suggest a potential role for co-culture with tumor CAFs in promoting tumor cell metastasis and growth, as suggested by other studies [53,54]. Co-culture with CAF positively affects organoid growth and metastasis. We identified genes that could affect multidrug resistance in co-culture. Subsequently, groups involved in interferon-alpha/beta signaling and MHC class II protein complex assemblies were identified.

Our findings show that activation of immune-related genes, such as interferon, which are known to belong to the antiviral pathway in CRC organoids, results in TLR3 activation in cancer cells in response to chemotherapy, highlighting the role of the TME in this innate immune response [55].

Interferon alpha (IFNα), a member of the type I IFN family, is well-recognized for its antiviral activities and is significant in cancer biology, as evidenced by our findings and existing reports [56]. In head and neck squamous-cell carcinoma and inflammatory breast cancer, TME-induced IFNα/β signaling activates the JAK/STAT signaling pathway, further contributing to DNA damage resistance [57,58].

MHC-II is a heterodimer comprising alpha and beta chains. MHC proteins are highly polymorphic, allowing for a diversity of peptides present in the population [59]. There are five isotypes of the class II HLA protein designated as HLA-DM, -DO, -DP, -DQ, and -DR [60]. HLA-DM is mainly regulated with HLA-DR, making our results relevant [61]. The expression of MHC-II and its associated machinery is primarily regulated by the class II transactivator (CIITA), induced by IFNγ through the JAK/STAT pathway, involving JAK1, JAK2, and STAT1 [61,62,63]. The functional role of MHC-II molecules, particularly in cancer, is an active area of research. Although traditionally associated with professional antigen-presenting cells (APCs), recent reports indicate that epithelial cells can express MHC-II, functioning as non-professional APCs in mucosal areas. This expression is critical for maintaining immune tolerance and surveillance [64,65,66,67]. Furthermore, the presence of MHC-II may promote immune tolerance through mechanisms such as T cell anergy or induction of Treg differentiation [68,69]. Cancer cells may exploit MHC-II expression to evade immune detection.

We infer that IFNα/β signaling and MHC class complexes are organically connected. CAFs secrete soluble factors that increase CRC chemoresistance after exposure to antimetabolites and DNA-damaging agents, such as 5-FU and oxaliplatin [70]. These factors, secreted by CAFs, can induce the activation of the PI3K/AKT/survivin and JAK/STAT pathways, which may protect against cell death, ensuring correct DNA repair and eventually induce resistance to oxaliplatin and 5-FU [71,72]. In this study, we identified two sets of gene groups potentially associated with resistance to DNA-damaging anticancer drugs through the JAK/STAT pathway by co-culturing with CAFs.

Although organoid-based co-culture models provide valuable insights, they may not fully capture the complexity and heterogeneity of in vivo tumor–stroma interactions. Additional clinical studies are needed to confirm our results, and functional studies related to the mechanisms of improved pharmacotherapy are necessary.

## 5. Conclusions

In conclusion, this study offers insights into the TME of CRC, highlighting the pivotal role of CAFs in influencing both tumor progression and chemoresistance.

Our findings demonstrated that the co-culture of CRC organoids with CAFs led to notable changes in genes specifically associated with IFNα/β signaling and MHC class II protein complex assembly. These pathways are crucial for modulating the immune response within the TME, thereby affecting the tumor response to chemotherapy.

This study adds to the existing body of knowledge on TME and highlights the significance of CAFs in cancer progression and drug resistance. Enhancing our understanding of these interactions can lead to advancements in personalized medical research.

## Figures and Tables

**Table 1:** Critical points of drug-resistance biomarkers in patient-derived colorectal cancer organoid and fibroblast co-culture system.

Contents	Setup of Analysis	Methods	Objectives
Morphological analysis and characteristics of organoids and CAFs	Preparation of CRC organoids and CAFs; indirect co-culture using transwell system	Bright-field and immunofluorescence staining analysis	Analysis of organoid and CAF morphology; assessment of cell viability
Co-culture and transcriptome profiling	RNA extraction from co-cultured samples	RNA-seq analysis to determine gene expression profiles	Identification of differentially expressed genes (DEGs) in co-culture vs. monoculture
DEG profiling in CRC organoids in a co-culture	RNA-seq data processing using bioinformatics tools	DESeq2 used for differential gene expression analysis	DEGs related to drug resistance identified; comparison of gene expression between different treatment groups
Functional enrichment analysis of the significant genes in the co-culture and candidate gene selection	Filtering of significant genes based on expression levels (DEG) and functional relevance	Pathway analysis tools (e.g., GSEA, Metascape) used to determine functional relevance	Identification of key pathways (e.g., JAK/STAT signaling); functional categorization of DEGs and potential biomarkers for drug resistance identified

## Data Availability

The RNA-Seq data from this study were submitted to the NCBI Sequence Read Archive (SRA) under BioProject ID PRJNA1087446.

## Abbreviations

CAF	Cancer-associated fibroblast
CRC	Colorectal cancer
CIITA	Class II transactivator
DAPI	4′,6-Diamidino-2-phenylindole dihydrochloride
DEG	Differentially expressed gene
ECM	Extracellular matrix
FBS	Fetal bovine serum
FDR	False discovery rate
GO	Gene ontology
HCC	Hepatocellular carcinoma
HNSCC	Head and neck squamous-cell carcinoma
IBC	Inflammatory breast cancer
IFNα	Interferon alpha
MHC	Major histocompatibility complex
NGS	Next-generation sequencing
PDAC	Pancreatic ductal adenocarcinoma
PDO	Patient-derived organoid
PPI	Protein–protein interaction
RIN	RNA integrity number
TME	Tumor microenvironment
TPM	Transcripts per million
5-FU	5-Fluorouracil

## References

[1] Sung H., Ferlay J., Siegel R.L., Laversanne M., Soerjomataram I., Jemal A., Bray F. (2021). Global cancer statistics 2020: GLOBOCAN estimates of incidence and mortality worldwide for 36 cancers in 185 countries. CA A Cancer J. Clin..

[2] Siegel R.L., Miller K.D., Goding Sauer A., Fedewa S.A., Butterly L.F., Anderson J.C., Cercek A., Smith R.A., Jemal A. (2020). Colorectal cancer statistics, 2020. CA A Cancer J. Clin..

[3] Wong M.C., Huang J., Lok V., Wang J., Fung F., Ding H., Zheng Z.-J. (2021). Differences in incidence and mortality trends of colorectal cancer worldwide based on sex, age, and anatomic location. Clin. Gastroenterol. Hepatol..

[4] Goldberg R.M., Rothenberg M.L., Van Cutsem E., Benson A.B., Blanke C.D., Diasio R.B., Grothey A., Lenz H.-J., Meropol N.J., Ramanathan R.K. (2007). The continuum of care: A paradigm for the management of metastatic colorectal cancer. Oncologist.

[5] Hurwitz H., Fehrenbacher L., Novotny W., Cartwright T., Hainsworth J., Heim W., Berlin J., Baron A., Griffing S., Holmgren E. (2004). Bevacizumab plus irinotecan, fluorouracil, and leucovorin for metastatic colorectal cancer. N. Engl. J. Med..

[6] Maughan T.S., James R.D., Kerr D.J., Ledermann J., McArdle C., Seymour M., Cohen D., Hopwood P., Johnston C., Stephens R.J. (2002). Comparison of survival, palliation, and quality of life with three chemotherapy regimens in metastatic colorectal cancer: A multicentre randomised trial. Lancet.

[7] Moodley Y., Govender K., van Wyk J., Reddy S., Ning Y., Wexner S., Stopforth L., Bhadree S., Naidoo V., Kader S. (2023). Predictors of treatment refusal in patients with colorectal cancer: A systematic review. Seminars in Oncology.

[8] Demissie K., Oluwole O.O., Balasubramanian B.A., Osinubi O.O., August D., Rhoads G.G. (2004). Racial differences in the treatment of colorectal cancer: A comparison of surgical and radiation therapy between Whites and Blacks. Ann. Epidemiol..

[9] Kaltenmeier C., Malik J., Yazdani H., Geller D.A., Medich D., Zureikat A., Tohme S. (2020). Refusal of cancer-directed treatment by colon cancer patients: Risk factors and survival outcomes. Am. J. Surg..

[10] Shin Y.M., Han H.S., Lim S.W., Kim B.C., Cheoi K.S., Eum Y.O., Kim S.T., Lee K.H. (2005). Combination chemotherapy of oxaliplatin, 5-fluorouracil and low dose leucovorin in patients with advanced colorectal cancer. Cancer Res. Treat. Off. J. Korean Cancer Assoc..

[11] Yaffee P., Osipov A., Tan C., Tuli R., Hendifar A. (2015). Review of systemic therapies for locally advanced and metastatic rectal cancer. J. Gastrointest. Oncol..

[12] Kim H.K., Choi I.J., Kim C.G., Kim H.S., Oshima A., Michalowski A., Green J.E. (2011). A gene expression signature of acquired chemoresistance to cisplatin and fluorouracil combination chemotherapy in gastric cancer patients. PLoS ONE.

[13] Bhowmick N.A., Neilson E.G., Moses H.L. (2004). Stromal fibroblasts in cancer initiation and progression. Nature.

[14] Sahai E., Astsaturov I., Cukierman E., DeNardo D.G., Egeblad M., Evans R.M., Fearon D., Greten F.R., Hingorani S.R., Hunter T. (2020). A framework for advancing our understanding of cancer-associated fibroblasts. Nat. Rev. Cancer.

[15] Vermeulen L., De Sousa E Melo F., Van Der Heijden M., Cameron K., De Jong J.H., Borovski T., Tuynman J.B., Todaro M., Merz C., Rodermond H. (2010). Wnt activity defines colon cancer stem cells and is regulated by the microenvironment. Nat. Cell Biol..

[16] Malanchi I., Santamaria-Martínez A., Susanto E., Peng H., Lehr H.-A., Delaloye J.-F., Huelsken J. (2012). Interactions between cancer stem cells and their niche govern metastatic colonization. Nature.

[17] Orimo A., Gupta P.B., Sgroi D.C., Arenzana-Seisdedos F., Delaunay T., Naeem R., Carey V.J., Richardson A.L., Weinberg R.A. (2005). Stromal fibroblasts present in invasive human breast carcinomas promote tumor growth and angiogenesis through elevated SDF-1/CXCL12 secretion. Cell.

[18] O’Connell J.T., Sugimoto H., Cooke V.G., MacDonald B.A., Mehta A.I., LeBleu V.S., Dewar R., Rocha R.M., Brentani R.R., Resnick M.B. (2011). VEGF-A and Tenascin-C produced by S100A4+ stromal cells are important for metastatic colonization. Proc. Natl. Acad. Sci. USA.

[19] De Palma M., Biziato D., Petrova T.V. (2017). Microenvironmental regulation of tumour angiogenesis. Nat. Rev. Cancer.

[20] Barrett R.L., Puré E. (2020). Cancer-associated fibroblasts and their influence on tumor immunity and immunotherapy. eLife.

[21] Nazareth M.R., Broderick L., Simpson-Abelson M.R., Kelleher R.J., Yokota S.J., Bankert R.B. (2007). Characterization of human lung tumor-associated fibroblasts and their ability to modulate the activation of tumor-associated T cells. J. Immunol..

[22] Tauriello D.V., Palomo-Ponce S., Stork D., Berenguer-Llergo A., Badia-Ramentol J., Iglesias M., Sevillano M., Ibiza S., Cañellas A., Hernando-Momblona X. (2018). TGFβ drives immune evasion in genetically reconstituted colon cancer metastasis. Nature.

[23] Salmon H., Franciszkiewicz K., Damotte D., Dieu-Nosjean M.-C., Validire P., Trautmann A., Mami-Chouaib F., Donnadieu E. (2012). Matrix architecture defines the preferential localization and migration of T cells into the stroma of human lung tumors. J. Clin. Investig..

[24] Gandellini P., Andriani F., Merlino G., D’Aiuto F., Roz L., Callari M. (2015). Complexity in the tumour microenvironment: Cancer associated fibroblast gene expression patterns identify both common and unique features of tumour-stroma crosstalk across cancer types. Semin. Cancer Biol..

[25] Peng S., Chen D., Cai J., Yuan Z., Huang B., Li Y., Wang H., Luo Q., Kuang Y., Liang W. (2021). Enhancing cancer-associated fibroblast fatty acid catabolism within a metabolically challenging tumor microenvironment drives colon cancer peritoneal metastasis. Mol. Oncol..

[26] Luo X., Fong E.L.S., Zhu C., Lin Q.X.X., Xiong M., Li A., Li T., Benoukraf T., Yu H., Liu S. (2021). Hydrogel-based colorectal cancer organoid co-culture models. Acta Biomater..

[27] Liu J., Li P., Wang L., Li M., Ge Z., Noordam L., Lieshout R., Verstegen M.M., Ma B., Su J. (2021). Cancer-associated fibroblasts provide a stromal niche for liver cancer organoids that confers trophic effects and therapy resistance. Cell. Mol. Gastroenterol. Hepatol..

[28] Xiao W., Pahlavanneshan M., Eun C.-Y., Zhang X., DeKalb C., Mahgoub B., Knaneh-Monem H., Shah S., Sohrabi A., Seidlits S.K. (2022). Matrix stiffness mediates pancreatic cancer chemoresistance through induction of exosome hypersecretion in a cancer associated fibroblasts-tumor organoid biomimetic model. Matrix Biol. Plus.

[29] Senthebane D.A., Rowe A., Thomford N.E., Shipanga H., Munro D., Al Mazeedi M.A., Almazyadi H.A., Kallmeyer K., Dandara C., Pepper M.S. (2017). The role of tumor microenvironment in chemoresistance: To survive, keep your enemies closer. Int. J. Mol. Sci..

[30] Senthebane D.A., Jonker T., Rowe A., Thomford N.E., Munro D., Dandara C., Wonkam A., Govender D., Calder B., Soares N.C. (2018). The role of tumor microenvironment in chemoresistance: 3D extracellular matrices as accomplices. Int. J. Mol. Sci..

[31] Devarasetty M., Mazzocchi A.R., Skardal A. (2018). Applications of bioengineered 3D tissue and tumor organoids in drug development and precision medicine: Current and future. BioDrugs.

[32] Neal J.T., Li X., Zhu J., Giangarra V., Grzeskowiak C.L., Ju J., Liu I.H., Chiou S.-H., Salahudeen A.A., Smith A.R. (2018). Organoid modeling of the tumor immune microenvironment. Cell.

[33] Skardal A., Devarasetty M., Rodman C., Atala A., Soker S. (2015). Liver-tumor hybrid organoids for modeling tumor growth and drug response in vitro. Ann. Biomed. Eng..

[34] Lee C., Hong S.N., Kim E.R., Chang D.K., Kim Y.H. (2021). Epithelial regeneration ability of Crohn’s disease assessed using patient-derived intestinal organoids. Int. J. Mol. Sci..

[35] Lee C., Song J.H., Cha Y.E., Chang D.K., Kim Y.H., Hong S.N. (2022). Intestinal epithelial responses to IL-17 in adult stem cell-derived human intestinal organoids. J. Crohn’s Colitis.

[36] Han Y.H., Ryu K.B., Medina Jiménez B.I., Kim J., Lee H.Y., Cho S.J. (2020). Muscular development in urechis unicinctus (Echiura, Annelida). Int. J. Mol. Sci..

[37] Kim D., Langmead B., Salzberg S.L. (2015). HISAT: A fast spliced aligner with low memory requirements. Nat. Methods.

[38] Pertea M., Pertea G.M., Antonescu C.M., Chang T.-C., Mendell J.T., Salzberg S.L. (2015). StringTie enables improved reconstruction of a transcriptome from RNA-seq reads. Nat. Biotechnol..

[39] Pertea M., Kim D., Pertea G.M., Leek J.T., Salzberg S.L. (2016). Transcript-level expression analysis of RNA-seq experiments with HISAT, StringTie and Ballgown. Nat. Protoc..

[40] Yu G., Wang L.-G., Han Y., He Q.-Y. (2012). clusterProfiler: An R package for comparing biological themes among gene clusters. Omics A J. Integr. Biol..

[41] Zhou Y., Zhou B., Pache L., Chang M., Khodabakhshi A.H., Tanaseichuk O., Benner C., Chanda S.K. (2019). Metascape provides a biologist-oriented resource for the analysis of systems-level datasets. Nat. Commun..

[42] Li T., Wernersson R., Hansen R.B., Horn H., Mercer J., Slodkowicz G., Workman C.T., Rigina O., Rapacki K., Stærfeldt H.H. (2017). A scored human protein–protein interaction network to catalyze genomic interpretation. Nat. Methods.

[43] Türei D., Korcsmáros T., Saez-Rodriguez J. (2016). OmniPath: Guidelines and gateway for literature-curated signaling pathway resources. Nat. Methods.

[44] Chatr-Aryamontri A., Oughtred R., Boucher L., Rust J., Chang C., Kolas N.K., O’Donnell L., Oster S., Theesfeld C., Sellam A. (2017). The BioGRID interaction database: 2017 update. Nucleic Acids Res..

[45] Szklarczyk D., Kirsch R., Koutrouli M., Nastou K., Mehryary F., Hachilif R., Gable A.L., Fang T., Doncheva N.T., Pyysalo S. (2023). The STRING database in 2023: Protein–protein association networks and functional enrichment analyses for any sequenced genome of interest. Nucleic Acids Res..

[46] Snel B., Lehmann G., Bork P., Huynen M.A. (2000). STRING: A web-server to retrieve and display the repeatedly occurring neighbourhood of a gene. Nucleic Acids Res..

[47] Kamali Zonouzi S., Pezeshki P., Razi S., Rezaei N. (2021). Cancer-associated fibroblasts in colorectal cancer. Clin. Transl. Oncol..

[48] Jin M.-Z., Jin W.-L. (2020). The updated landscape of tumor microenvironment and drug repurposing. Signal Transduct. Target. Ther..

[49] Wu T., Dai Y. (2017). Tumor microenvironment and therapeutic response. Cancer Lett..

[50] Chen X., Song E. (2019). Turning foes to friends: Targeting cancer-associated fibroblasts. Nat. Rev. Drug Discov..

[51] Bu L., Baba H., Yasuda T., Uchihara T., Ishimoto T. (2020). Functional diversity of cancer-associated fibroblasts in modulating drug resistance. Cancer Sci..

[52] Chiavarina B., Turtoi A. (2017). Collaborative and defensive fibroblasts in tumor progression and therapy resistance. Curr. Med. Chem..

[53] Khan I., Steeg P.S. (2021). Endocytosis: A pivotal pathway for regulating metastasis. Br. J. Cancer.

[54] Kajiho H., Kajiho Y., Frittoli E., Confalonieri S., Bertalot G., Viale G., Di Fiore P.P., Oldani A., Garre M., Beznoussenko G.V. (2016). RAB2A controls MT1-MMP endocytic and E-cadherin polarized Golgi trafficking to promote invasive breast cancer programs. EMBO Rep..

[55] Vitiello G.A.F., Ferreira W.A.S., Cordeiro de Lima V.C., Medina T.D.S. (2021). Antiviral responses in cancer: Boosting antitumor immunity through activation of interferon pathway in the tumor microenvironment. Front. Immunol..

[56] Rizza P., Moretti F., Belardelli F. (2010). Recent advances on the immunomodulatory effects of IFN-α: Implications for cancer immunotherapy and autoimmunity. Autoimmunity.

[57] Hosein A.N., Livingstone J., Buchanan M., Reid J.F., Hallett M., Basik M. (2015). A functional in vitro model of heterotypic interactions reveals a role for interferon-positive carcinoma associated fibroblasts in breast cancer. BMC Cancer.

[58] Ma H., Yang W., Zhang L., Liu S., Zhao M., Zhou G., Wang L., Jin S., Zhang Z., Hu J. (2019). Interferon-alpha promotes immunosuppression through IFNAR1/STAT1 signalling in head and neck squamous cell carcinoma. Br. J. Cancer.

[59] Trowsdale J. (1993). Genomic structure and function in the MHC. Trends Genet..

[60] Unanue E.R., Turk V., Neefjes J. (2016). Variations in MHC class II antigen processing and presentation in health and disease. Annu. Rev. Immunol..

[61] Thibodeau J., Bourgeois-Daigneault M.-C., Lapointe R. (2012). Targeting the MHC Class II antigen presentation pathway in cancer immunotherapy. Oncoimmunology.

[62] Van Den Elsen P.J. (2011). Expression regulation of major histocompatibility complex class I and class II encoding genes. Front. Immunol..

[63] Axelrod M.L., Cook R.S., Johnson D.B., Balko J.M. (2019). Biological consequences of MHC-II expression by tumor cells in cancer. Clin. Cancer Res..

[64] Beyaz S., Chung C., Mou H., Bauer-Rowe K.E., Xifaras M.E., Ergin I., Dohnalova L., Biton M., Shekhar K., Eskiocak O. (2021). Dietary suppression of MHC class II expression in intestinal epithelial cells enhances intestinal tumorigenesis. Cell Stem Cell.

[65] Rescigno M., Lopatin U., Chieppa M. (2008). Interactions among dendritic cells, macrophages, and epithelial cells in the gut: Implications for immune tolerance. Curr. Opin. Immunol..

[66] Biton M., Haber A.L., Rogel N., Burgin G., Beyaz S., Schnell A., Ashenberg O., Su C.-W., Smillie C., Shekhar K. (2018). T helper cell cytokines modulate intestinal stem cell renewal and differentiation. Cell.

[67] Vellano C.P., White M.G., Andrews M.C., Chelvanambi M., Witt R.G., Daniele J.R., Titus M., McQuade J.L., Conforti F., Burton E.M. (2022). Androgen receptor blockade promotes response to BRAF/MEK-targeted therapy. Nature.

[68] Nadafi R., de Graça C.G., Keuning E.D., Koning J.J., de Kivit S., Konijn T., Henri S., Borst J., Reijmers R.M., van Baarsen L.G. (2020). Lymph node stromal cells generate antigen-specific regulatory T cells and control autoreactive T and B cell responses. Cell Rep..

[69] Lei P.-J., Pereira E.R., Andersson P., Amoozgar Z., Van Wijnbergen J.W., O’Melia M.J., Zhou H., Chatterjee S., Ho W.W., Posada J.M. (2023). Cancer cell plasticity and MHC-II–mediated immune tolerance promote breast cancer metastasis to lymph nodes. J. Exp. Med..

[70] Sethy C., Kundu C.N. (2021). 5-Fluorouracil (5-FU) resistance and the new strategy to enhance the sensitivity against cancer: Implication of DNA repair inhibition. Biomed. Pharmacother..

[71] Gonçalves-Ribeiro S., Díaz-Maroto N., Berdiel-Acer M., Soriano A., Guardiola J., Martínez-Villacampa M., Salazar R., Capella G., Villanueva A., Martínez-Balibrea E. (2016). Carcinoma-associated fibroblasts affect sensitivity to oxaliplatin and 5FU in colorectal cancer cells. Oncotarget.

[72] Gu J., Li Z., Zhou J., Sun Z., Bai C. (2019). Response prediction to oxaliplatin plus 5-fluorouracil chemotherapy in patients with colorectal cancer using a four-protein immunohistochemical model. Oncol. Lett..

